# In vitro and in vivo evaluation of a photosensitive polyimide thin-film microelectrode array suitable for epiretinal stimulation

**DOI:** 10.1186/1743-0003-10-48

**Published:** 2013-05-29

**Authors:** Xia Jiang, Xiaohong Sui, Yiliang Lu, Yan Yan, Chuanqing Zhou, Liming Li, Qiushi Ren, Xinyu Chai

**Affiliations:** 1 School of Biomedical Engineering, Shanghai Jiao Tong University, 800 Dongchuan Road 200240, Shanghai, China; 2 Department of Biomedical Engineering, College of Engineering, Peking University, Beijing, China

**Keywords:** Photosensitive polyimide, Microelectrode, Epiretinal, Retinal prostheses, Biocompatibility, Electrophysiology

## Abstract

**Background:**

Epiretinal implants based on microelectro-mechanical system (MEMS) technology with a polyimide (PI) material are being proposed for application. Many kinds of non-photosensitive PIs have good biocompatibility and stability as typical MEMS materials for implantable electrodes. However, the effects of MEMS microfabrication, sterilization and implantation using a photosensitive polyimide (PSPI) microelectrode array for epiretinal electrical stimulation has not been extensively examined.

**Methods:**

A novel PSPI (Durimide 7510) microelectrode array for epiretinal electrical stimulation was designed, fabricated based on MEMS processing and microfabrication techniques. The biocompatibility of our new microelectrode was tested in vitro using an MTT assay and direct contact tests between the microelectrode surface and cells. Electrochemical impedance characteristics were tested based on a three-electrode testing method. The reliability and stability was evaluated by a chronic implantation of a non-functional array within the rabbit eye. Histological examination and SEM were performed to monitor possible damage of the retina and microelectrodes. Electrically evoked potentials (EEPs) were recorded during the acute stimulation of the retina.

**Results:**

The substrate was made of PSPI and the electrode material was platinum (Pt). The PSPI microelectrode array showed good biocompatibility and appropriate impedance characteristics for epiretinal stimulation. After a 6-month epiretinal implantation in the eyes of rabbits, we found no local retinal toxicity and no mechanical compression caused by the array. The Pt electrodes adhesion to the PSPI remained stable. A response to electrical stimuli was with recording electrodes lying on the visual cortex.

**Conclusion:**

We provide a relevant design and fundamental characteristics of a PSPI microelectrode array. Strong evidences on testing indicate that implantation is safe in terms of mechanical pressure and biocompatibility of PSPI microelectrode arrays on the retina. The dual-layer process we used proffers considerable advantages over the more traditional single-layer approach and can accommodate much many electrode sites. This lays the groundwork for a future, high-resolution retinal prosthesis with many more electrode sites based on the flexible PSPI thin film substrate.

## Background

The recent development of epiretinal prosthetic implants has progressed rapidly owing to the production of thin-film flexible microelectrode arrays based on micro-electro-mechanical system (MEMS) processing techniques [1-6]. The positioning of the epiretinal prostheses and the retinal anatomy and physiology means that these prostheses have properties and functions that differ somewhat from other neural (retinal) implants. Epiretinal prostheses are placed directly on the internal limiting membrane (ILM) and the ganglion cell layer of retina [7-12]. The microelectrodes electrically stimulate the retinal ganglion cells directly, initiating an action potential that must be delivered to the cortex for an evoked visual response. The retina is an exceptionally soft and fragile tissue and increases the difficulty of epiretinal surgery and prosthesis implantation. An appropriate stiffness in the construction of the prosthesis is essential to aid the implantation during surgery and so that the prosthesis will closely contact the ganglion layer and take the shape of retina without retinal compression. However, overly increasing the stiffness of the prosthesis will increase the mechanical pressure on the retina and may result in tissue damage. As a special neural implant, the microelectrodes should also be safe and acceptable for long-term usage.

Polyimide (PI) is the most frequently used substrate material for the manufacture of bio-MEMS epiretinal microelectrodes. There are traditional non-photosensitive PIs that use a photoresist mask as an etching template in one of the standard steps. Compared to non-photosensitive PIs, novel photosensitive PIs (PSPIs) can be patterned directly by UV light and developer chemicals without the use of photoresist layer. This can provide a highly reliable microelectrode array with fewer manufacturing steps. Many of the bio-MEMS applications that have employed non-photosensitive PIs have been shown to have good biocompatibility as an implantable medical device [13-16]. However, a systematic evaluation of PSPI (Durimide 7510) thin-film microelectrode arrays has not been reported for use as epiretinal implants. Sun et al. studied the cytotoxicity of PSPI - Durimide 7020 in vitro [17], while Stieglitz et al. compared the long-term stability in vitro and conventional biocompatibility of different PIs including a PSPI (Durimide 7510) [16,19]. They found that these PSPIs did not elicit any toxic response in cell culture testing. Myllymaa et al. investigated surface characterization and in vitro biocompatibility of the PSPI- PI-2771 [18]. The electrochemical properties of PSPI epiretinal microelectrodes or electrophysiological characterization are also unknown. Information about the reliability, sterilization, and biocompatibility of PSPI-film electrodes that will be implanted on the surface of retina needs to be systematically collected.

Prosthetic arrays must be evaluated in vitro and in vivo prior to clinical trials. According to the requirements for electrical stimulation of the retina, we designed a novel MEMS-based microelectrode array with dual metal layer using PSPI as a substrate material instead of traditional non-photosensitive polyimide or parylene. This paper summarizes the biocompatibility, fundamental design, and evaluation of our PSPI thin-film microelectrode array for epiretinal electrical stimulation, with emphasis on the characteristics that are suitable for implantation in the eye.

## Methods

### MEMS-processing and microfabrication

**Figure 1:**
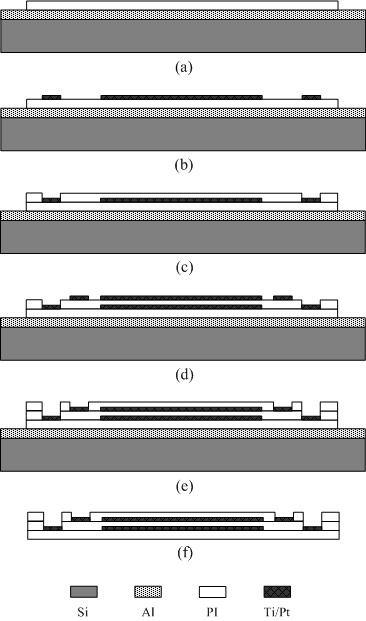
**The manufacturing procedures of dual metal-layer microelectrode array.** (**a**) Evaporation of aluminum sacrificial layer onto the silicon wafer and spin-coating of the first PSPI film; (**b**) Sputtering and patterning of the lower Ti/Pt layer; (**c**) Spin-coating and patterning of the middle PSPI layer to expose half of the stimulating electrodes and bonding pads; (**d**) Sputtering and patterning upper Ti/Pt layer; (**e**) Spin-coating and patterning of the upper PSPI layer to expose the whole stimulating electrodes and bonding pads; (**f**) Microelectrode array released from the silicon substrate.

The implantable flexible multichannel microelectrode array was fabricated based on the PSPI Durimide 7510 (Arch Chemicals, Norwalk, CT, USA). The fabrication process of the PSPI-based microelectrode array is shown in Figure 1. A 1 μm thick aluminum coating was deposited on a one side polished silicon wafer (46th Institute of Electronic Science and Technology, Tianjin, China) cleaned by standard RCA criteria, and dried at 200°C. This was used as a sacrificial layer for releasing the structure. A 20 μm thick lower PSPI layer was spin-coated onto the silicon wafer, patterned, and heated in a nitrogen atmosphere to 350°C. A metallic film of Ti/Pt (100 Å/3000 Å) was sputtered and patterned using the “lift-off” processing technique, and this layer used as interconnecting lines between the electrodes and bonding pads. Then, a middle 5 μm thick PSPI layer was spin-coated onto the lower metal layer. Photolithography was used to directly expose half of the stimulating electrodes and bonding pads due to the photosensitivity of the PSPI. The upper Ti/Pt patterned film was obtained by the same method. After that, a 5 μm thick PSPI upper layer was added to encapsulate the electrodes and photolithography used to expose the total number of stimulating electrodes and bonding pads. Finally, the polyimide microelectrode array was released from the silicon substrate by electrochemical erosion. The wafer was immersed in a 20%; NaCl solution and a DC power supply of 0.7 – 0.8 V applied to the anodic wafer by a copper clip and a cathodic platinum wire; about 40 h is necessary to release a 7.6 cm silicon wafer. The aluminum sacrificial layer can be successively etched due to the electrical conductivity of the silicon wafer with a relatively low resistivity of 7.83 - 10.58 ***Ω***•cm.

### PSPI-based electrodes layout

**Figure 2:**
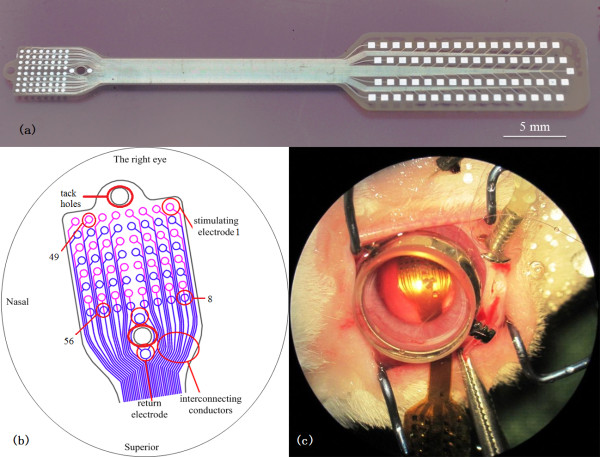
**Prototype of the 64-channel dual-metal-layer thin-film microelectrode array.** (**a**) The whole array structure; (**b**) expanded view of the stimulating electrodes implanted on the surface of rabbit retina (Numbers 1, 8,49,56 were indicated the electrodes used for the Figure 8 in the section on EEPs); (**c**) placement of the stimulating array in the rabbit eye.

The 64 8 × 8 stimulating electrodes (each electrode 300 μm in diameter) subtended an area of approximately 4.7 × 4.7 mm (inter-electrode space of 250 μm) (Figure 2). The curve of the outer shape of the array was selected for its adaptation to the retinal curvature and ease of implantation. The interconnecting conductors were 30 μm wide and the space between columns was 40 μm. Two 700 μm diameter holes were incorporated into the design so that titanium nails could be used to stabilize the array in-vivo. Two 400 μm diameter electrodes were placed on either of one of the titanium (Ti) tack holes and used as return electrodes with the Ti nail. A buffer layer of titanium was used to reinforce the bonding of electrodes on the substrate. The squared bonding pads were 500 μm squares. The lower 32 (8 × 4) and the other upper 32 (8 × 4) metal stimulating electrodes were alternatively distributed. To reduce the neural damage resulting from the electrode array implantation, the curved corners were utilized.

### In vitro testing

#### Sterilization and cytotoxicity of the PSPI-based electrodes array

The microelectrode array samples were cleaned with the deionized water and autoclaved for 3 cycles of 20 min at 121°C in a stainless steel Vertical Autoclave (DSX-280B, Shanghai, China) to achieve the desired sterility assurance level. The biocompatibility and cytotoxicity was tested in vitro according to ISO 10993 (ISO 10993–5 2009) protocols. Mouse L929 cells (American Type Culture Collection CCL, NCTC clone 929) were cultured and used for quantitative measurements (e.g. proliferation, cell survival). The tetrazolium salt 3-(4, 5-dimethylthiazol-2-yl)-2, 5-diphenyltetrazolium bromide (MTT) assay was performed according to the method of Mosmann and Zange [20,21]. Cells were observed using an inverted light microscope (OLYMPUS, CKX41) or scanning electron microscopy (HITACHI, S-520). Changes in general morphology, vacuolization, detachment, cell lysis, and membrane integrity were assessed and compared with a blank (sample not exposed to extracts) of fresh and stained cells. Cytotoxicity was determined according to the scale based on ISO 10993–5 standards. Nine microelectrode samples were assessed in this test.

In the first kind of assay, L929 cells were exposed to cells were exposed to a solution containing any soluble elements that may have dissolved from the array. Each solution was prepared in accordance with ISO 10993–12 protocol. Six 1 cm^2^ pieces per milliliter of cell culture medium were used and incubated for 24 h at 37°C. The MTT assay was used to measure cell viability and proliferation in an ELISA reader (TECAN, Salzburg, Austria) at a wavelength of 570 nm (test) and 690 nm (reference). L929 cells were plated into 96-well microtiter plates (CELLSTAR®) at a density of 5 × 10^3^ cells/well. After 24 h, culture medium was replaced by 100 ***μ***l of the test solution, the negative control, or the blank, and incubated for 12, 24, 48, 72, 96, or 120 h (n = 3 per time). The media was refreshed every 48 h. The MTT solution (20 μl; 5 mg MTT/ml phosphate-buffered saline, Sigma) was added to each well, followed by 4 hours incubation in the dark. Then the MTT solution decanted and 150 μl of dimethyl sulfoxide (DMSO, Gibco) added. A microplate reader was used to measure the spectrophotometric absorbance. The cell culture media was Dulbecco’s Modified Eagle Medium (DMEM, Gibco) supplemented with 10%; (v/v) heat-inactivated fetal calf serum (Gibco) and the antibiotics penicillin/streptomycin (500×), lyophilisate (100 IU/ml ) and streptomycin (100 μg/ml). A fresh culture medium served as negative control. All test samples and the negative control materials were sterilized in an autoclave (121°C, 20 min).

The second cytotoxic assay was based on direct contact tests between the microelectrode surface and the cells. The array was directly placed on the cell layer and incubated for 3, 6, 24, or 72 h at 37 C, 95%; relative humidity in an air atmosphere containing 5%; CO_2_ (n = 3 per time). Cells were observed using inverted light microscope (OLYMPUS, CKX41) and scanning electron microscopy (HITACHI, S-520). Changes in general morphology, vacuolization, detachment, cell lysis, and membrane integrity were assessed and compared with a blank (cell samples not exposed to extracts). Cytotoxicity was determined according to the following equation based on ISO 10993–5 standard to calculate the reduction of viability compared to the blank.

$$\mathrm{Viab}.\%;=100\times {\mathrm{OD}}_{570e}/{\mathrm{OD}}_{570b}$$

*OD*_*570e*_ was the mean value of the measured optical density of the test sample, and *OD*_*570b*_ is the mean value of the measured optical density of the blanks. The lower the Viab.%; value, the higher the cytotoxic potential of the test item is. If viability is reduced to < 70%; of the blank, it has a cytotoxic potential.

### Electrochemical characterization

The electrochemical characterization included cyclic voltammetry (CV) and electrical impedance spectrometry (EIS). Cyclic voltamentry is a common method used to evaluate electrochemical characteristics. A commercial electrochemical testing system (ZAHNER Im6 electrochemical workstation, Germany) was used to acquire the cyclic voltammogram (−800 to 800 mV at 30 mV/s) and calculate the charge storage capacity of the electrodes.

Electrochemical impedance of the sterilized array was tested in-vitro in a phosphate buffered saline (PBS) solution. The testing work was carried out based on a precision LCR meter (Agilent E4980A, Agilent Technologies, Santa Clara, CA) and three-electrode testing equipment including a working electrode, a large flat platinum counter electrode, and a Ag/AgCl reference electrode. An AC voltage of 50 mV_pp_ was applied. Ten microelectrode arrays were tested.

### In vivo testing

#### Implantation

Three healthy adult Chinese albino rabbits (Fengxian, Shanghai, China), each weighing 2.0 to 2.5 kg, were used in the experiments. All the experimental procedures were conducted in accordance with the ARVO Statement for the Use of Animals in Ophthalmic and Vision Research and the policies in the Guide to the Care and Use of Laboratory Animals issued by the US National Institutes of Health and were approved by the Ethics Committee of Shanghai Jiao Tong University *Surgical procedures*. Three electrode arrays were cleaned and sterilized as detailed above. The rabbits were anesthetized by intravenous injection of a 5%; pentobarbital sodium (Pentobarbital sodium, Urchem Ltd, Shanghai, China) at an initial dose of 5 mg/kg, and were maintained with the dose of 15 mg/kg/h. Electrocardiograms and respiration rate were monitored throughout the experiments (MPA 2000, ALCBIO Ltd., Shanghai, China). Body temperature was kept at 39°C by a temperature controller (H-KWDY-III; Xinxiaoyuan Biotech Ltd., Nanjing, China). A standard three-port vitrectomy through the pars plana area was carried out in the right eye, leaving the left eye as a control. Briefly, a sclerotomy was made 1 mm behind the sclerocorneal limbus at 8 o’clock position by using a 20 G sclerotome, and a cannula sutured in place for irrigation. Then, two other sclerotomies were made, one at the 10 position and the other at 2 o’clock for the insertion of an optical fiber and vitrectomy probe into the vitreous cavity. Then a standard pars plana vitrectomy was performed, after which the vitreous was completely removing and the sclerotomy enlarged to the width of the array. The electrode array film was then inserted into the cavity and fixed at the surface of the retina by titanium tacks (Figure 2).

### Histology and scanning electron microscope (SEM)

Postoperatively, the rabbits were treated with prednisolone acetate 1%; eye drops (Yanlijiang Ltd., Hangzhou, China) five times daily for one week. Eye examinations were performed before the operation, then at 2, 4, 6, and 24 w after the surgery, with recording the appearance of the implanted site and observing cornea, pupil, sclera, conjunctiva and pupillary response to light. The eyeballs of three implanted rabbits were taken out after the acute stimulation and at the end of 6th and 24th weeks, respectively. They were fixed in 10%; neutral-buffered formalin solution at room temperature. The retinas were stripped with the removal of the pigment layer. The retinas taken from the area under the implant were dehydrated with graded alcohol solutions, cleared in xylene, embedded in paraffin and cut into 5 μm slices in a plane perpendicular to the tack position and then stained with hematoxylin-eosin (HE). The implanted electrodes were fixed in glutaraldehyde for electron microscope scanning in order to evaluate the interface between the implant and the inner limiting membrane. Implant sites were examined for inflammation, necrosis, fibroplasia, fibrosis and fatty infiltrates. Local effects after epiretinal implantation were determined according to an irritant ranking score: non-irritant (0–2.9) compared to the negative controls, based on ISO 10993–6 standard. An experience pathologist examined them by using an objective score system (ISO 10993–6:2007, Annex E), in the microscopic field at a magnification of × 400.

### Acute stimulation of the retina

Three animals were anaesthetized and the retinal prosthesis implanted as described above. The pupils were dilated by tropicamide (Tropicamide-DCPC, Double Cranes Pharmaceutical Co., Ltd., Beijing, China) and neosynephrine (Adrenaline Hydrochloride Injection, Harvest Pharmaceutical Co., Ltd., Shanghai, China). The animal dark-adapted for 30 min. The electrode array film was fixed on the surface of retina over the macula area, and return electrode positioned in the sclera. The skull was exposed through a skin incision along the midline, and two trephine holes drilled over the visual cortex contralateral to the stimulated eye (5 mm anterior to the lambdoid suture and 4 mm lateral to the midline, and 3 mm posterior to the first hole). Two screw-type stainless steel electrodes were screwed into the holes to contact the dura mater, and were used for recording the electrically evoked potentials (EEPs) in the cortex. A screw-type stainless steel electrode was used a contralateral reference electrode placed 6 mm anterior to the bregma suture and 4 mm lateral to the midline. A stainless steel needle electrode was placed subcutaneously in the ear tip as the ground electrode. Retinal stimulation consisted of a 50 μA 1 Hz symmetrical charge balanced cathodic first biphasic pulse (pulse width- 0.5 ms), generated by an isolated and programmable current source stimulator (MS16, Tucker-Davis Technologies, Alachua, FL, USA); Various pulse intensities, widths, frequencies and stimulating waveform were tested. Prior to the acute electrical stimulation, an in vivo impedance test was implemented as outlined in the in vitro methods. The impedances of the electrodes were measured with an applied AC voltage of 50 mV_PP_ and frequencies ranging from 20 to 100 kHz.

### Statistical analysis

The results of the MTT test and histology and the impedance testing were analyzed statistically using ANOVA test at a significance level of *P* < 0.05. Statistical analysis were performed with SPSS 15.0 and Microsoft EXCEL 2010 software.

## Results

### PSPI-based microelectrode array and epiretinal implantation

The prosthesis interfaces with the inner limiting membrane of the epiretinal surface. Patient and skilled surgery procedures were required with the epiretinal placement because of softness of thin PSPI film. There was a temporary fluid collection in the space along the inserted array after the operation; however, the fluid was absorbed during the first post-operative week. There were no definite post-operative complications.

### In vitro

#### Cytotoxicity of PSPI-based electrodes array

The cell viability showed the cytotoxicity results for our microelectrode arrays made of PSPI and Pt when added to L929 mouse fibroblast cells in vitro and determined using the MTT assay. The viability of the test solution were 96.16%;, 78.60%;, 81.66%;, 95.56%;, 99.02%; and 90.43%;, respectively at the different time. It is clear that cell death occurred in the cultures over the five day culture period, however, there were no significant differences between the experimental and control samples (*P* < 0.05). This indicates that the array materials were nontoxic to the cultured cells.

**Figure 3:**
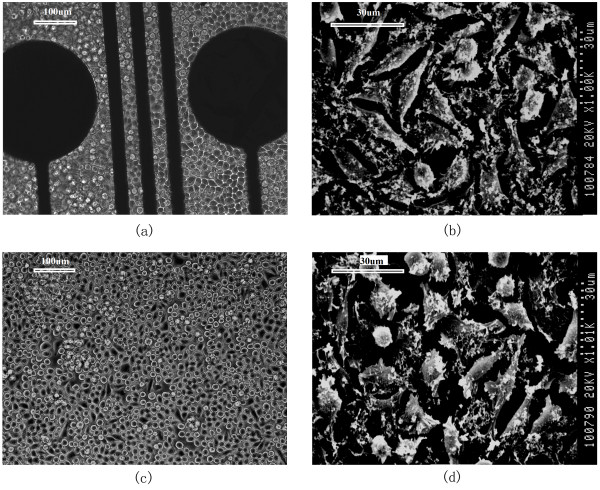
**Morphology of the L929 cells growing directly on the microelectrode.** (**a** and **b**) Cells were photographed with an inverted light microscope (× 200) or by scanning electron microscopy, respectively after 3 days in culture. (**c** and **d**) Control cultures under the same conditions.

When L929 fibroblast cells were co-cultured with the array they readily attached to the array surface and spread out on electrode film after 12 h incubation. These L929 cells grew quickly over a 24 h incubation period and had numerous cellular extensions. The highest density of cells was observed on day 5, and cells exhibited a normal fibroblastic morphology (Figure 3).

#### Impedance and CV of PSPI-based electrodes

**Figure 4:**
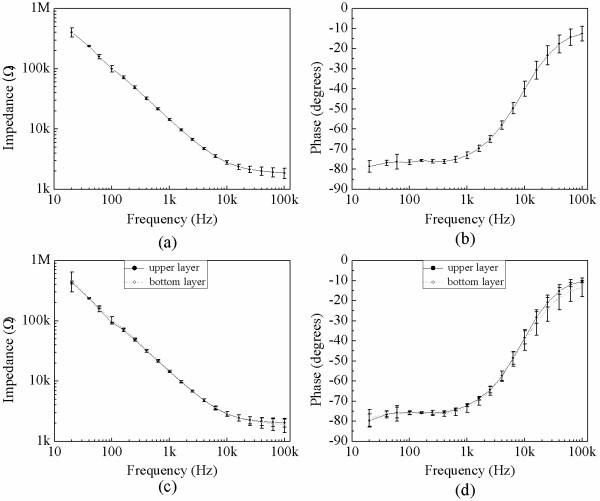
**Morphology of the L929 cells growing directly on the microelectrode.** Impedance (**a**) and phase (**b**) for all electrodes (n = 3); impedance (**c**) and phase (**d**) for the upper and bottom layers of the same arrays.

Three thin-film electrode arrays were randomly selected and tested. Figure 4 shows that the magnitude of the impedance decreased as frequency increased, and a lower resistance, especially at high frequencies (> 1 kHz). The good high-pass characteristic of the array was consistent with other electrode impedances described in the literature [22]. The phase angle also decreased with increasing frequency, showing more obvious interface properties about double-layer capacitance. The value of phase angles gradually stabilized at frequencies higher than 1.5 kHz and was close to zero at extremely high frequencies, and thus showed no obvious interface properties.

The average electrode impedance at 1 kHz was 14.47 ± 0.52 k***Ω*** and the average phase angle was −73.06 ± 1.60 degrees. Because the electrode was fabricated by a double-layer MEMS process, we also evaluated the electrical properties of the two electrode layers separately. Figures 4c and d show the average impedances and phases of the electrodes on both layers of one array. The impedances of both layers were not significantly different. The average impedances of the bottom and upper electrode layers were 14.45 ± 0.33 k***Ω*** and 14.75 ± 0.74 k***Ω*** at 1 kHz, respectively. The absolute values of the phase angle of electrodes on both layers changed as the frequency increased, but were not significantly different. The phase angles of the bottom and upper electrodes were −73.34 ± 2.16 degrees and −72.16 ± 0.49 degrees respectively, when the frequency was 1 kHz. Our results indicated that the electrical properties of the upper and lower layers manufactured with double-process multi-channel processing were highly consistent.

**Figure 5:**
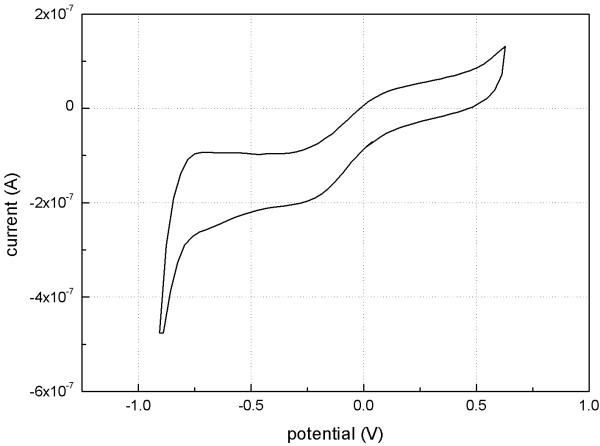
**Cyclic voltammogram of the electrodes.** It was at a 30 mV/s sweep rate in physiological saline solution.

The current–voltage curve without redox peaks indicated that the charge was transferred mainly through the charging and discharging of the capacitive double layers of the microelectrodes. The charge-transferring pattern ensures the security of the microelectrodes and avoids irreversible electrochemical reactions. A cyclic voltammogram is shown in Figure 5. The charge storage capacity was 2.808 mC/cm^2^, showing a high charge storage capability and the effective transfer of the charge.

### In vivo

#### Chronic retinal implants

**Figure 6:**
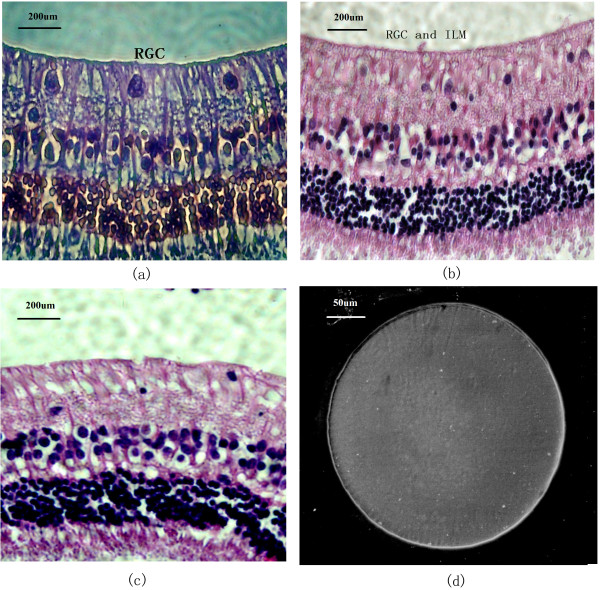
**The morphology of the inner retina layer and microelectrode arrays surface after implantation.** (**a**) Morphology of the inner retinal layer after the microelectrode was implanted for six weeks (retinal ganglion cell layer, RGC; inner limiting membrane, ILM). (phase contrast microscope, 400 ×); (**b**): rabbit retina layer six months after implant. (light microscope, 400 ×); (**c**): control retina. (**d**): SEM of a microelectrode six months after the implant; the electrode showed no damage to the surface or accumulation of tissue matter.

The biocompatibility of the array and the long term effects of implantation on the array were evaluated following the chronic implantation (up to six months) of a non-functional array. The arrays were classified as non-irritants without significant difference between test retinas and negative controls (P < 0.05; 2.7 and 0, respectively, i.e. both classed as non-irritant based on the ISO 10993–6 standards) following a six month implantation. As shown in Figure 6a-c, there was no evidence of hypertrophy, atrophy, dystrophy or cell degeneration caused by mechanical pressure or material cytotoxicity. The results indicate that implantation of the thin and flexible PSPI sheet of electrodes did not harm the inner retina or cause changes to the retinal layers. In addition, neither the sterilization process nor the implant environment damaged or changed the array. Light microscopy and SEM showed no cracks in the PSPI layer and no delamination of the metal electrodes was observed and electrodes and interconnecting conductors were still adhering to the durimide substrate (Figure 6d).

### In vivo impedance testing

**Figure 7:**
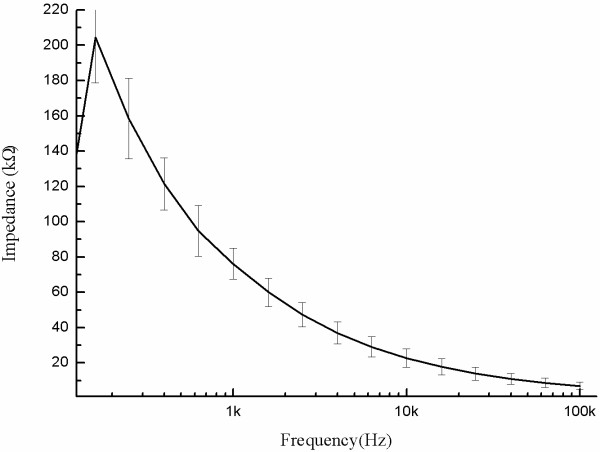
**The average impedance magnitude of in vivo electrodes.** Impedance for all electrodes in the three implanted arrays are shown.

Prior to the acute animal experiments, an in vivo impedance testing of the three electrode arrays was implemented. The average electrode impedance in vivo at 1 kHz was 64.25 ± 10.46 k***Ω***, and was higher than that measured in vitro (14.47 ± 0.52 k***Ω***, *P* < 0.05). However, the electrode impedance and resistance decreased with an increase in stimulus frequency, which is similar to the in vitro test, especially at high frequencies (> 1 kHz Figure 7).

### Electrically evoked cortical activation by the microelectrode

**Figure 8:**
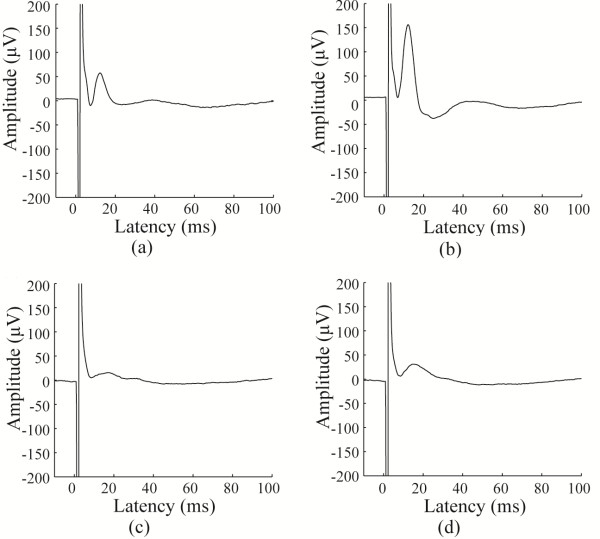
**Typical EEPs elicited by electrical stimulation of rabbit retina using a 0.5 ms 50** ***μ*A current pulse.** EEPs could be recorded following simultaneous stimulation by channels 1–8 (**a**, **b**) and channels 49–56 (**c**, **d**). (**a** and **b**) show the difference in EEP potential amplitude between the anterior and posterior recording electrodes, respectively. The same convention applies to (**c**) and (**d**). The channels in the stimulating electrode arrays were indicated in Figure 2b.

The in-vivo electrophysiology experiments were performed to confirm that cortical activation occurred after retinal stimulation with array. Typical EEPs elicited by electrical current stimulation are shown in Figure 8. When the upper-nasal side of the retina was stimulated by a column of eight electrodes with current intensity of 50 μA, the evoked response amplitude was higher at the posterior recording electrodes. When stimulating the lower-temporal side of the retina by another column of electrodes, the cortical response could be recorded with lower amplitude.

## Discussion

In order to design an ideal epiretinal microelectrode, we must consider challenges that stem from biology, medicine, electrical and mechanical engineering, and the chemical properties of each component. We designed, fabricated, and tested a thin-film PSPI-based epiretinal microelectrode array, with an emphasis on the feasibility of it being implanted onto the surface of the neural retinal. Notable research by the different epiretinal prosthesis groups have developed various prostheses, such as the Argus I 16-channel and Argus II 60-channel microelectrode array [7,9], the 49-channel Intelligent Retinal Implant System™ [10], and the 25-channel EPI-RET array [12, see also 11]. Most of these microelectrodes are based on traditional non-photosensitive polyimide due to its desirable mechanical properties and biocompatibility. As a novel polyimide, PSPI has some technological advantages in the current area of bio-MEMS. Rousche et al. [23] designed and fabricated PSPI based cortical electrodes. As a recording devise, Myllymaa et al. employed PSPI (PI-2711) as an encapsulating layer in flexible microelectrode arrays that were capable of recording rat cortical EEPs [24] and Spence et al. [25] used flexible multielectrodes based on Photoneece PWDC-1000 to resolve multiple muscles in an insect appendage. Durimide 7510 is one of the biologically compatible PSPIs and we chose it to be the substrate for thin-film electrodes for epiretinal stimulation. Photopatternability simplifies the electrode manufacturing process of lithography and etching to expose the electrode sites of epiretinal implants. This advantage is much more evident in the fabrication of multi-metal-layer electrode arrays. It is also desirable due to its lower cost process with improved yields.

Electrodes should be capable of delivering sufficient electrical current within safe charge injection limits. Previously, platinum or a platinum-iridium alloy was widely used for neural stimulating electrodes, due to their charge injection capacity and long-term durability of platinum [5,22,26,27]. Although the security limits of titanium nitride are higher than platinum and iridium as stimulating electrodes, a lower survival rate of retinal cells was reported in biocompatibility experiments [28]. We chose platinum as the material for stimulating electrode, as it is one of the most commonly used materials for visual prostheses [9-12]. In our study, the Pt electrode interface impedance in vitro was controlled within 20 k***Ω*** or less (room temperature, 1 kHz, 50 mVpp). The impedance magnitude in vivo was larger than that in vitro, which is due to the higher resistivity of the biological tissue. Several considerations need to be taken into account to reduce the electrode impedance magnitudes. First, a solution widely used to decrease impedance is to increase the effective surface area of the electrode, for example, as electroplating, surface roughening, and chemical modification. Second, some other fabrication materials can be chosen, such as TiN, to modify the surface of Pt electrodes. These methods will be considered in the microfabrication of novel MEMS microelectrode arrays in our future research. Although it may be beneficial to have the array surface adhere well to host proteins, the absorption of a thick layer of protein can reduce the sensitivity/conductance of the electrodes. Thus the effect of protein adherence may play an important role in the performance of electrodes, and should be considered when developing in vivo chronic neural stimulation. Our SEM analysis showed that there was very little tissue adhering to the array surface after chronic implantation. However, the long-term effects of protein absorption on electrochemical properties of stimulating electrodes need more data.

Some hurdles need to be overcome in the design and development process of an epiretinal prosthesis before commercial realization. Biocompatibility is the basic requirement of neural implants and considered by some to be of primary importance [29]. The MTT assay indicated that, according to ISO-109935 guidelines, Durimide 7510 is non-cytotoxic there were no apparent deleterious effects on cell viability and is in agreement with the recent paper of Stieglitz et al. [19]. Currently, three PSPIs have been shown to be non-toxic biomaterials for neural in vitro implants [17-19]. The ILM forms a barrier between the retina and the vitreous [30] and is the interface between the epiretinal prosthesis and retinal tissue. Retinal ganglion cells will atrophy or degenerate in response to malnutrition caused by mechanical pressure of electrode array [31] and glial cells, including astrocytes and Mueller cells, then hypertrophy in response to injury. The retinal stimulating electrode must have the flexibility to match the retinal curvature without producing significant mechanical pressure at the retinal surface but at the same time must be stiff enough to be easily handled and positioned during surgery. There is only about a 3 mm space between the termination of the retina and the insertion of the ciliary body [30]. This is the preferred surgical approach for epiretinal implantation because the sclera can be incised without damaging the retina or ciliary body. However, the length of the surgical incision limits the width of the array and its interconnecting lines. This was overcome by using a dual metal-layer arrangement, which reduced the space needed for the 64 stimulating lines and the returning electrodes. Because the stimulating and returning electrodes were on the same substrate of the thin-film array, an additional returning electrode in the vitreous body was eliminated. This design plus the flexibility and rounded edges of our PSPI electrode arrays reduced the damage to the retina and made our implantation easier and more convenient. The array must also be biocompatible with the host tissue and must avoid environmental aging and de-adhesion of the polyimide-mental films. The lack of retinal tissue damage after a six month implantation period in vivo are in agreement with previous work [16,19], and the lack of changes to the array properties and structural integrity confirmed the suitability of our design and potential clinical application of the PSPI-film electrode array.

One of the most important factors for researchers is the basic function of epiretinal electrical stimulation. Our array occupied an area 5 mm in diameter, less than the overall geometry of the human macular region. A retinal stimulating array ideally needs to pack at least 1000 electrodes into this 5 × 5 mm space for ideal resolution [32]. The dual-layer process proffers considerable advantages over the more traditional single-layer approach [33] in that it can accommodate many more electrode sites for higher resolution. A new high density electrode array based on a parylene substrate was designed and fabricated with 1024 platinum electrodes [34], but no systematic in vivo ocular evaluation on the safety and electrophysiology of the whole electrode array has been reported. The actual cortical response and resolution for such a retinal prosthesis is also unknown. The substrate material for epiretinal stimulating electrode arrays serves as a mechanical carrier and electrical insulator, which is a key component assuring the safe and effective signal transmission to the underlying tissues. Electrical stimulation in our acute experiments showed that the array functioned effectively in situ. Detailed information on the electrophysiological properties and long-term effects of electrical stimulation after implantation need further exploration.

## Conclusion

Recent PI-MEMS techniques for epiretinal electrical stimulation have provided new advances in this direction [32,34-36]. The substrates used are traditional non-photosensitive PI. In this paper, we provided a relevant design and fundamental characteristics of a PSPI microelectrode array. In vivo and in vitro results show that the array is safe in terms of mechanical pressure and biocompatibility. This lays the ground work for a future, high-resolution retinal prosthesis with many more electrode sites using multi-layer MEMS techniques based on the flexible PSPI thin film substrate.

## Abbreviations

DMEM: Dulbecco’s Modified Eagle Medium; MEMS: Microelectro-mechanical system; MTT: Tetrazolium salt 3-(4, 5-dimethylthiazol-2-yl)-2, 5-diphenyltetrazolium bromide; PI: Polyimide; PSPI: Photosensitive polyimide; SEM: Scanning electron microscope.

## Competing interests

The authors declare that they have no competing interests.

## Authors’ contributions

XC, XJ and XS designed the test and carried out the analysis of data. XC and XS fabricated the electrodes and tested the impedance. XJ performed the biocompatibility tests in vitro and in vivo. XJ and XS wrote the manuscript. All authors were active in the editing process of the manuscript. All authors read and approved the final manuscript.
